# Hidden Variables in Deep Learning Digital Pathology and Their Potential to Cause Batch Effects: Prediction Model Study

**DOI:** 10.2196/23436

**Published:** 2021-02-02

**Authors:** Max Schmitt, Roman Christoph Maron, Achim Hekler, Albrecht Stenzinger, Axel Hauschild, Michael Weichenthal, Markus Tiemann, Dieter Krahl, Heinz Kutzner, Jochen Sven Utikal, Sebastian Haferkamp, Jakob Nikolas Kather, Frederick Klauschen, Eva Krieghoff-Henning, Stefan Fröhling, Christof von Kalle, Titus Josef Brinker

**Affiliations:** 1 Digital Biomarkers for Oncology Group National Center for Tumor Diseases German Cancer Research Center (DKFZ) Heidelberg Germany; 2 Institute of Pathology University Hospital Heidelberg University of Heidelberg Heidelberg Germany; 3 Department of Dermatology University Hospital Kiel University of Kiel Kiel Germany; 4 Institute for Hematopathology Hamburg Hamburg Germany; 5 Private Institute of Dermatopathology Heidelberg Germany; 6 Private Institute of Dermatopathology Friedrichshafen Germany; 7 Skin Cancer Unit German Cancer Research Center (DKFZ) Heidelberg Germany; 8 Department of Dermatology University Medical Center Mannheim University of Heidelberg Mannheim Germany; 9 Department of Dermatology University Hospital of Regensburg Regensburg Germany; 10 Department of Medicine III RWTH University Hospital Aachen Aachen Germany; 11 Institute of Pathology Charité University Hospital Berlin Berlin Germany; 12 National Center for Tumor Diseases German Cancer Research Center (DKFZ) Heidelberg Germany; 13 Department of Clinical-Translational Sciences Charité and Berlin Institute of Health Berlin Germany

**Keywords:** artificial intelligence, machine learning, deep learning, neural networks, convolutional neural networks, pathology, clinical pathology, digital pathology, pitfalls, artifacts

## Abstract

**Background:**

An increasing number of studies within digital pathology show the potential of artificial intelligence (AI) to diagnose cancer using histological whole slide images, which requires large and diverse data sets. While diversification may result in more generalizable AI-based systems, it can also introduce hidden variables. If neural networks are able to distinguish/learn hidden variables, these variables can introduce batch effects that compromise the accuracy of classification systems.

**Objective:**

The objective of the study was to analyze the learnability of an exemplary selection of hidden variables (patient age, slide preparation date, slide origin, and scanner type) that are commonly found in whole slide image data sets in digital pathology and could create batch effects.

**Methods:**

We trained four separate convolutional neural networks (CNNs) to learn four variables using a data set of digitized whole slide melanoma images from five different institutes. For robustness, each CNN training and evaluation run was repeated multiple times, and a variable was only considered learnable if the lower bound of the 95% confidence interval of its mean balanced accuracy was above 50.0%.

**Results:**

A mean balanced accuracy above 50.0% was achieved for all four tasks, even when considering the lower bound of the 95% confidence interval. Performance between tasks showed wide variation, ranging from 56.1% (slide preparation date) to 100% (slide origin).

**Conclusions:**

Because all of the analyzed hidden variables are learnable, they have the potential to create batch effects in dermatopathology data sets, which negatively affect AI-based classification systems. Practitioners should be aware of these and similar pitfalls when developing and evaluating such systems and address these and potentially other batch effect variables in their data sets through sufficient data set stratification.

## Introduction

The advent of artificial intelligence (AI) in digital pathology (DP) has resulted in the development of various algorithms for the detection, classification, and further evaluation of multiple cancer subtypes [1]. General interest and enthusiasm for this emerging technology continues to grow, exemplified by the development of a variety of convolutional neural network (CNN)–based oncology systems for the analysis of histological images of breast [2,3], lung [4], skin [5,6], and gastrointestinal [7] cancer. However, the successful implementation of CNN-based assistance systems in DP is complicated by a plethora of challenges [8-10], some of which are domain-specific, while others are omnipresent in the field of deep learning (DL) and machine learning (ML) in general.

**Figure 1:**
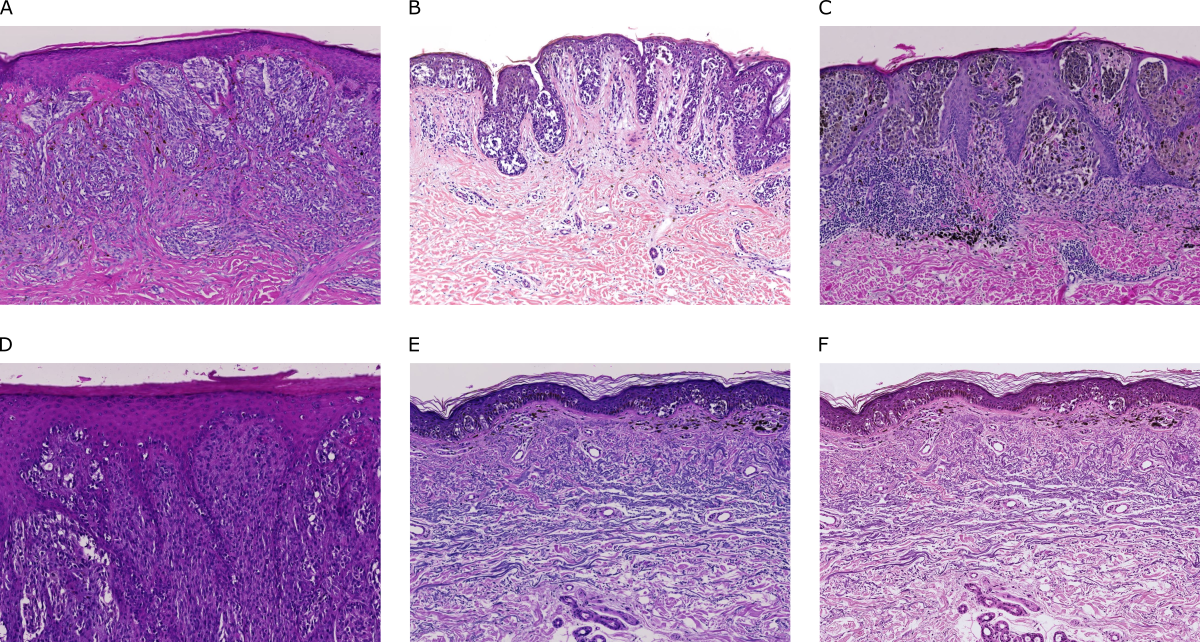
Comparison of exemplary whole slide image sections obtained at different institutes: (A) Institute of Pathology, University Hospital Heidelberg, University of Heidelberg, Heidelberg, Germany (Zeiss scanner; Carl Zeiss AG); (B) Department of Dermatology, University Hospital Kiel, University of Kiel, Kiel, Germany (3DHISTECH scanner; 3DHISTECH Ltd); (C) Private Institute of Dermatopathology, Mönchhofstraße 52, Heidelberg, Germany (Zeiss scanner); (D) Department of Dermatology, University Medical Center Mannheim, University of Heidelberg, Mannheim, Germany (Zeiss scanner); and (E) Private Institute of Dermatopathology, Siemensstraße 6/1, Friedrichshafen, Germany (Zeiss scanner). (F) The same slide section as shown in (E) but scanned with a Hamamatsu scanner (Hamamatsu Photonics KK) rather than a Zeiss scanner.

One important issue in the field of biomedical data analysis is the occurrence of batch effects, which are defined as differences among subsets of a data set introduced through technological artifacts [11,12]. In DP, such artifacts are introduced during tissue processing and slide preparation [13], and presumably also during slide digitization, image compression, and storage [14], all of which affect slide and image appearance (Figure 1). We expand on this definition of batch effects by including biological factors, presumably unrelated to the actual classification task, as causative agents. Both factors (biological and nonbiological) are referred to as hidden variables from here on.

Batch effects can be problematic during development of ML models, where hidden variables are learned instead of or in addition to the intended target variables. The hidden variable then acts as a complete or partial proxy for the intended target variable, negatively affecting the model’s performance. Studies have addressed this issue by focusing on various normalization techniques [15-19]. In addition, standardized preprocessing procedures and balanced data set construction may aid in reducing but not eliminating batch effects. Overall, this is concerning, and a previous study using a breast cancer tissue cohort has already suggested the existence of batch effects in parts of the publicly available Cancer Genome Atlas (TCGA) pathology repository [20].

We expand on these findings by analyzing the learnability of four exemplary selected hidden variables, as learnable variables can cause batch effects that can negatively influence DL algorithms. Our research on hidden variables aims to highlight that batch effects are not an unlikely occurrence, thereby reinforcing the importance of proper data set construction and experimental design, as well as sensitizing the community toward these and similar pitfalls for the emerging field of DL in DP.

## Methods

### Study Design

Using a proprietary dermatopathological data set of anonymized slides, a series of ML tasks were formulated, where each task investigated the learnability of a certain hidden variable. The analyzed variables are believed to be found throughout DP whole slide image (WSI) data sets, but their learnability does not necessarily need to generalize.

Next, multiple DL models of the same architecture were trained on the variable stated by each task, followed by subsequent performance analysis, where an assessment was made of whether the task’s variable was learnable or unlearnable. A variable was considered learnable when the 95% confidence interval of its mean balanced accuracy had a lower bound above 50.0% when calculated on the slide level. Note that a random classifier, which is unable to learn the variable, would be expected to achieve a balanced accuracy of approximately 50.0%.

Ethics approval was obtained from the ethics committee of the Medical Faculty of Mannheim, University of Heidelberg, Mannheim, Germany.

### Data Set

A total of 427 hematoxylin and eosin–stained preparations were obtained from five different institutes, with each slide belonging to one patient and containing tissue sections of melanoma biopsies (Table 1). For details on the slide digitization process, see Multimedia Appendix 1.

**Table 1:** Overview of individual data sets.

Data set	Origin of slides	Number of slides	Number of tiles	Scanner type	Tasks
1	Heidelberg^a^	81	1,344,825	Zeiss^b^	2 (slide preparation date); 3 (slide origin)
2	Kiel^c^	196	2,092,726	3DHISTECH^d^	1 (patient age); 2 (slide preparation date)
3	Heidelberg^e^	73	832,940	Zeiss	3 (slide origin)
4	Friedrichshafen^f^	54	350,518; 364,196	Zeiss; Hamamatsu^g^	3 (slide origin); 4 (scanner type)
5	Mannheim^h^	23	513,256	Zeiss	3 (slide origin)

^a^Institute of Pathology, University Hospital Heidelberg, University of Heidelberg, Heidelberg, Germany.

^b^Carl Zeiss AG.

^c^Department of Dermatology, University Hospital Kiel, University of Kiel, Kiel, Germany.

^d^3DHISTECH Ltd.

^e^Private Institute of Dermatopathology, Mönchhofstraße 52, Heidelberg, Germany.

^f^Private Institute of Dermatopathology, Siemensstraße 6/1, Friedrichshafen, Germany.

^g^Hamamatsu Photonics KK.

^h^Department of Dermatology, University Medical Center Mannheim, University of Heidelberg, Mannheim, Germany.

### Classification Tasks

Four classification tasks were performed, each analyzing one predefined hidden variable. Data sets for each task were chosen based on data availability, while simultaneously minimizing the risk of cross-task learning. For instance, all data sets were used for the slide origin prediction task (task 3) except data set 2, as the Department of Dermatology, University Hospital Kiel, used a different scanner type (3DHISTECH scanner; 3DHISTECH Ltd) to digitize the slides (Table 1). Therefore, a classifier could potentially determine the slide origin for data set 2 by determining the scanner type.

#### Task 1: Patient Age

To determine patient age, data set 2 was used, and only slides with an assigned patient age were analyzed. Slides were divided into one of two classes based on patient age (≤48 years versus >78 years), excluding slides of patients with ages in between. The cutoff points were chosen in an effort to achieve a natural balance between both age groups, with the 30-year gap making it plausible to observe possible distinct age-dependent morphological features.

#### Task 2: Slide Preparation Date

To determine the year of slide preparation, data sets 1 and 2 were used. Data availability varied but was generally sufficient for years 2014-2018. For each data set, separate binary classification tasks were defined, where slides were taken from every other year to ensure that there was a minimum of 365 days between the preparation dates of slides from each class (eg, data set 1, 2015 versus 2017). This resulted in five separate classification subtasks.

#### Task 3: Slide Origin

To determine the origin of the respective slide, all data sets except data set 2 were used. Origin was defined as the institution from which the slides were obtained.

#### Task 4: Scanner Type

To determine the scanner type, data set 4 was used. These slides were scanned twice, but due to the slight difference in resolution between the Zeiss (Carl Zeiss AG) and Hamamatsu (Hamamatsu Photonics KK) scanners, scanned Zeiss slides were reprocessed specifically for this task by downscaling their resolution (0.22 µm/px) to match the resolution of the Hamamatsu scanner (0.23 µm/px).

### Model Training

Each task had a designated combined data set based on the setup outlined above. Each combined data set was divided into a training set and a test set on slide level using an 80:20 split. If the resulting test set contained fewer than 10 slides, a cross-validation approach on slide level was employed to increase the size of the test set to a minimum of 10 slides per class.

A ResNet50 architecture was trained for each task. In cases where cross-validation was used for testing, the number of trained CNNs equaled the number of cross-validation folds. The training run for each task was repeated a total of five times to obtain a robust average performance uninfluenced by stochastic training events. This number was chosen arbitrarily but with the intention to reduce the overall computing time. For exact technical details on the cross-validation and training procedure, please see Multimedia Appendix 1 or refer to Multimedia Appendix 2 for an exemplary jupyter notebook demonstrating the basic training procedure.

### Model Inference and Statistical Evaluation

Inference was carried out on each task’s respective test set using the complete set of tiles for each slide. The class for a WSI was computed by first predicting on its complete set of tiles, averaging all output probabilities, and assigning the class label with the highest average probability to the slide. Because each training and evaluation run for a task was repeated five times, a mean balanced accuracy with a corresponding 95% confidence interval could be computed.

## Results

Learnability was investigated on the slide level, as that is the standard and decisive criterion in DP. For all tasks, balanced accuracy was generally higher on slide level than on tile level.

For each task, a balanced accuracy over 50.0% was achieved, even when taking into account the range of the corresponding confidence intervals. Classifier performance varied widely inter-task and intra-task for task 2, which had multiple subtasks. Task 1 (patient age) had a mean balanced accuracy of 87.5% (Table 2). For task 2 (slide preparation date), performance varied widely between subtasks, ranging from 56.1% (95% CI 52.7% to 59.5%) to 83.5% (95% CI 80.9% to 86.1%). Classifiers for task 3 (slide origin) and 4 (scanner type) showed balanced accuracies of 97.9% (95% CI 97.3% to 98.5%) and 100%, respectively.

**Table 2:** Overall mean performance of each task’s classifiers measured using mean balanced accuracy and evaluated on tile level and slide level.

Task^a^		ResNet50 performance (mean balanced accuracy)	
		Tile level	Slide level (95% CI)^b^
1: Patient age		76.2%	87.5%
2: Slide preparation date			
	Data set 1: 2015 versus 2017	54.1%	56.1% (52.7% to 59.5%)
	Data set 1: 2016 versus 2018	56.5%	63.2% (53.4% to 73.0%)
	Data set 2: 2014 versus 2016	69.0%	82.0% (76.4% to 87.6%)
	Data set 2: 2015 versus 2017	66.6%	83.5% (80.9% to 86.1%)
	Data set 2: 2016 versus 2018	52.7%	56.7% (52.6% to 60.7%)
3: Slide origin		94.2%	97.9% (97.3% to 98.5%)
4: Scanner type		100%	100%

^a^Test sets for each task had a minimum of 10 slides per class.

^b^Confidence intervals are shown for the decisive criteria (slide level) and are omitted for tasks where no variation on slide level was observed.

**Figure 2:**
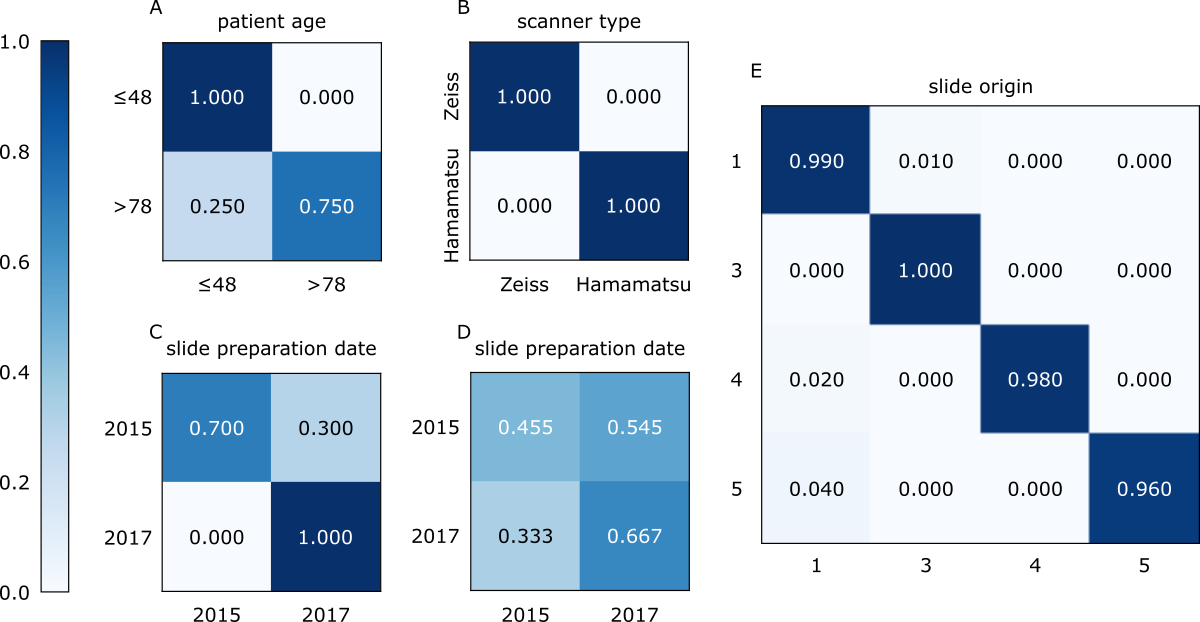
Distributions of the models’ predictions for tasks 1 to 4 on slide level for ResNet50 (run 1). (A) Task 1 (patient age) prediction. (B) Task 4 (scanner type) prediction. (C, D) Comparison of the two most distinct classification subtasks for task 2 (slide preparation date), where balanced accuracy was either at a maximum (C: data set 2, 2015 versus 2017) or at a minimum (D: data set 1, 2015 versus 2017). (E) Task 3 (slide origin) prediction, with the data set number displayed rather than the data set origin for better readability.

Looking at the distributions of each task-specific model for the first run, slide origin and scanner type could be predicted with very high accuracy, with minor misclassification errors for task 3 (slide origin) and no misclassifications for task 4 (scanner type). For task 1, patient age below 48 years could be predicted with high accuracy, but one-quarter of the slides originating from older patients (>78 years) were erroneously classified as belonging to the younger age group. For task 2 (slide preparation date), the results varied widely between comparisons. The 2-year comparison with the highest balanced accuracy (data set 2, 2015 versus 2017) showed some misclassifications, with slides from 2015 occasionally being classified as 2017, whereas the task with the lowest balanced accuracy (data set 1, 2015 versus 2017) showed frequent misclassifications in both directions (Figure 2).

## Discussion

### Principle Findings

Using four exemplary hidden variables found in DP WSI data sets, we showed that these variables were learned by a DL algorithm for a dermatopathological data set. A learnable hidden variable may cause a batch effect, which can greatly affect the training of such algorithms if said variable is unintendedly picked up instead of or in addition to the intended target variable. We hypothesized that there would be hidden variables that would figure very prominently on the slides. These are more likely to be picked up and used by the algorithm to classify the slides and therefore pose the greatest threat to classification accuracy. To identify such “high risk” variables, we limited the amount of training the CNN received, using a standard architecture and a training procedure with little optimization and no training until convergence. This should result in only prominent variables being learned, which likely pose the greatest threat to a classifier’s accuracy, although an influence of factors that may be learned by a more extensive training procedure cannot be excluded.

All of the four variables tested (patient age, slide preparation date, slide origin, and scanner type) were learned by the classifier, albeit to different extents. The highest balanced accuracy was observed for task 4 (scanner type). For this task, slides from data set 4 were scanned twice with different devices but processed with the same image-processing pipeline, leaving differences in scanner type (eg, specific scanner hardware or software) as the only causative source of variations, which must be quite pronounced based on the high balanced accuracy.

Performances for tasks 3 (slide origin) and 4 (scanner type) were comparable. As all slides for task 3 were scanned and processed using the same scanner type and pipeline, digitization as the source of the observed batch effects can be ruled out. Therefore, the source of variation most likely stemmed from the slide preparation step, a complex process with lots of potential variables related to the sectioning, fixation, staining, and mounting procedures. Determining the origin of images was previously shown outside the field of DP, where a CNN correctly identified hospital systems based solely on chest radiographs [21].

The aforementioned slide preparation step was also a likely contributor to the “classifiability” of the slide preparation dates (task 2), together with slide aging itself (ie, small amounts of tissue and dye degradation). While slide aging is presumably a gradual process, the slide preparation step is expected to change more abruptly when institutions change the exact mode of slide preparation over time (eg, when a new staining protocol is introduced). This could explain the large variability in classifier performance for task 2, where in some cases the difference between two particular years could be identified much more accurately than between two other years at the same institution. Based on these results, the learnability of this task depends highly on the chosen year and data set, making the underlying variable less of a risk factor but still worthwhile considering.

The patient age prediction task was the only task that reflected a true “biological” difference between the analyzed groups. It is known that the texture of the skin changes as result of the aging process via a multitude of processes [22]. For instance, the amount of elastin and collagen decreases with time, which results in a restructuring of the fibrous tissue in deeper skin layers. On UV-exposed skin, additional similar effects may also be induced by photoaging. Moreover, some extent of biological variability regarding the aging process likely exists. This rather complex pattern of skin aging may explain why the ability of the CNN to separate the chosen age groups was not perfect. Performance would likely drop if the age gap of 30 years were to be reduced or if instead of WSIs showing both tumor and healthy skin, only tumor regions were regarded. Nonetheless, the classifier’s ability to pick up variables representing large age differences is important to consider, as an unequal patient age distribution is not unlikely to occur for certain medical DL objectives, especially since cancer incidence increases with age.

Based on these findings, it is not unlikely that the discussed variables may interfere with the generation of an accurate CNN-based classifier. Due to the technical nature of slide preparation date, slide origin, and scanner type, their learnability could generalize to other fields in DP, which may have to be investigated in further studies. The learnability of patient age, however, may be more specific to the field of dermatology. While patient age is known to have an impact on the skin, age-related differences may be much less prominent in other tissues.

### Prevention and Verification

In order to minimize the learning of batch effect variables, we suggest to balance any known batch effect variables during creation of the training data set, in addition to any normalization and preprocessing standardization. If easy-to-learn variables are equally balanced between classes, separation based on these variables should no longer result in a reduction of the training loss, thus losing its optimization value. In addition, large and diversified validation sets decrease the likelihood that unwanted correlations between batch effect variables and the intended biological variable exist and thereby can aid in uncovering whether the intended biological variable or some confounding hidden variable was learned.

Unfortunately, balancing training data sets for all potential batch effects is unfeasible. Even randomized clinical trials can only ever be balanced for a few features that are deemed crucial to the comparison in question. With time, additional knowledge accumulating both within and outside the field of AI-supported medicine may help researchers to clarify which potential influencing variables have to be taken into account for which tasks. In this regard, the future realization of more transparent AI-systems that facilitate both explainability and causability [23] would go a long way in helping practitioners to better assess the reliability of AI-systems through a better understanding of their decision-making process.

### Limitations

A major limitation is that the list of considered artifacts is not exhaustive. As numerous other potential confounders exist, some of which we are not yet aware of, complete coverage of all possible artifacts in one study is impossible and therefore has to be limited to a selection considered to be crucial. However, because of the black-box nature of DL algorithms, there is no proof to show what the model actually learned, meaning that any of the unaccounted for artifacts could by chance correlate with a task’s class distribution and be learned instead. This cannot be ruled out; however, by increasing validation set size and diversity, chances of these accidental training set correlations persisting through to the validation set decrease and should therefore be detected during the validation stage.

In this study, only results obtained with one DL architecture are shown. We therefore investigated two additional architectures (DenseNet121 and VGG16) and observed a similar trend (see Multimedia Appendix 1).

While learnability was only investigated on a dermatopathological data set in this study, some of the insights gained here may be transferable to other fields in DP. Moreover, this study did not intend to show exactly what variables can be learned but rather to show that unexpected variables can be learned.

### Conclusions

Our DL model was able to learn several potential batch effect variables with relative ease, a finding that is likely to be applicable to other DL models, too. Thus, our results highlight the importance of identifying and minimizing these effects, not only by normalization and preprocessing standardization but also by carefully constructing training and validation sets for DL classification tasks.

## Appendix

Multimedia Appendix 1 Additional methods and results.

Multimedia Appendix 2 Exemplary jupyter notebook.

## Abbreviations

AI: artificial intelligence
CNN: convolutional neural network
DL: deep learning
DP: digital pathology
ML: machine learning
TCGA: The Cancer Genome Atlas
WSI: whole slide image
