# Supplementary material for: Hidden Variables in Deep Learning Digital Pathology and Their Potential to Cause Batch Effects: Prediction Model Study
Source: J Med Internet Res. 2021 Feb 2;23(2):e23436. doi: 10.2196/23436 (PMC7886613; doi:10.2196/23436)
Supplement: Multimedia Appendix 1 [file jmir_v23i2e23436_app1.docx]

# Supplementary Online Materials

## Supplementary Materials A. Slide digitization

Slides from all datasets except dataset 2 were scanned at 20-fold magnification (0.22 μm/px) using a Zeiss Axio Scan.Z1 digital slide scanner (Carl Zeiss AG). In addition, dataset 4 was scanned at 40-fold magnification (0.23 μm/px) with a NanoZoomer S360 digital slide scanner (Hamamatsu Photonics K.K.). Slides from dataset 2 were only scanned using a Pannoramic 1000 digital slide scanner (3DHISTECH Ltd.) set to 20-fold magnification (0.24 μm/px). Digitized slides were automatically segmented into square tiles of 512x512 pixels.

## Supplementary Materials B. Cross-validation and training of the model

If a task’s test set had less than 10 slides per class, a cross-validation approach was used to increase overall test set size. The combined dataset for each task was split into 5-folds, resulting in five train and five test sets with an 80:20 distribution for each train and test set. As the dataset size, and subsequently also test set size varied from task to task, a varying number of folds were used for each task, but always enough to ensure the overall test set contained at least 10 slides per class.

To counteract the strong variation in size of tissue section on the slides, a fixed subset of tiles (n=1,000) was randomly sampled from each slide and used for training. Class imbalances were counteracted with a combined approach of up- and downsampling, where any class containing more tiles than a certain threshold (t=50,000) was downsampled to 50,000. Minority classes were upsampled to either match the majority class or 50,000 in case the majority class(es) exceeded the threshold.

We used three architectures, pre-trained on ImageNet (ResNet50, DenseNet121, VGG16). Training followed a pre-defined procedure shared for all architectures, which was optimized separately for each task using a validation set obtained from the task’s respective training set in an 80:20 split. After hyperparameter optimization, the final training run was carried out on the combined train and validation set.

Training of each convolutional neural network (CNN) was done using mostly feature extraction, meaning that only the last fully-connected layers (head) were trained while the previous layers (body), responsible for feature extraction, were kept fixed. Training followed Leslie Smith’s 1 cycle policy [1] and was carried out with a learning rate of 0.001. The number of epochs for each task was as follows:

- Task 1: 15 epochs followed by 10 epochs where all layers were trained using a differential learning rate between 1e-7 and 1e-5.
- Task 2: 5 epochs
- Task 3: 10 epochs
- Task 4: 4 epochs

All work was carried out in Python 3.7.3, using the OpenSlide Python 1.1.1 library for slide pre-processing, fastai 1.0 for model development and Scikit-learn 0.21 for evaluation and statistical analysis.

## Supplementary Materials C. Performance for different architectures

Training runs for DenseNet121 and VGG16 architectures were only repeated once to cut down the overall computing time and as we only investigated if a similar trend is reproducible across architectures.

*Table 2. Overall performance for each task’s classifier on tile- and slide-level for DenseNet121 and VGG16.* Performance was measured using balanced accuracy and was evaluated on tile- and on slide-level. Task 1-4 were prediction of patient age, slide preparation date, slide origin and scanner type respectively. Test-sets for each task had a minimum of 10 slides per class. Training runs for each task were only repeated once, hence no confidence intervals were calculated. D, dataset.

| **Task** | | | **DenseNet121 performance** | | **VGG16 performance** | |
| --- | --- | --- | --- | --- | --- | --- |
|  |  |  | **Tile-level** | **Slide-level** | **Tile-level** | **Slide-level** |
|  | | |  |  |  |  |
| 1 | | | 76.8% | 87.5% | 78.4% | 87.5% |
| 2 | D1 | 2015  vs.  2017 | 56.2% | 60.6% | 52.8% | 44.8% |
|  |  | 2016  vs.  2018 | 55.3% | 56.4% | 52.8% | 60.3% |
|  | D2 | 2014  vs.  2016 | 67.9% | 75.0% | 67.6% | 75.0% |
|  |  | 2015  vs.  2017 | 68.4% | 81.7% | 64.7% | 80.8% |
|  |  | 2016  vs.  2018 | 51.7% | 57.1% | 53.2% | 54.2% |
| 3 | | | 92.8% | 97.1% | 94.7% | 98.1% |
| 4 | | | 99.8% | 100% | 100% | 100% |

## References

1. Smith LN. A disciplined approach to neural network hyper-parameters: Part 1--learning rate, batch size, momentum, and weight decay. arXiv preprint arXiv:180309820 2018.
